# Exploring the Impact of 3-*O*-Methylquercetin on Wnt/β-Catenin Pathway Activity and Its Potential in Neural Processes

**DOI:** 10.3390/ph18111680

**Published:** 2025-11-06

**Authors:** Kamila Leichtweis, Danilo Predes, Marielly C. Mangelli, Hugo Mauricio, Barbara S. M. de Jesus, Clara F. Charlier, Raquel C. da Silva, Giselle F. Passos, Luiz F. S. Oliveira, Clara O. Nogueira, Samir F. A. Cavalcante, Diego M. Lopes, Rodrigo S. Almeida, Danielle C. Bonfim, Alessandro B. C. Simas, Julia R. Clarke, Pedro S. M. Pinheiro, Jose G. Abreu

**Affiliations:** 1 Instituto de Ciências Biomédicas, Universidade Federal do Rio de Janeiro, Rio de Janeiro 21941-902, Brazil; danilopredes@gmail.com (D.P.); mary_28carvalho@hotmail.com (M.C.M.); hugomauricio1999@gmail.com (H.M.); mascarenhas.barbi@gmail.com (B.S.M.d.J.); clara.forrer.charlier@gmail.com (C.F.C.); oliveira.lfs2@gmail.com (L.F.S.O.); clara.nogueira@hotmail.com (C.O.N.); danibom@gmail.com (D.C.B.); juclarke@gmail.com (J.R.C.); pedro.pinheiro@icb.ufrj.br (P.S.M.P.); 2 Faculdade de Farmácia, Universidade Federal do Rio de Janeiro, Rio de Janeiro 21941-853, Brazil; rcsilva1984@gmail.com (R.C.d.S.); gfazzioni@farmacia.ufrj.br (G.F.P.); 3 Instituto de Pesquisas de Produtos Naturais Walter Mors, Universidade Federal do Rio de Janeiro, Rio de Janeiro 21941-853, Brazil; samir.cavalcante@eb.mil.br (S.F.A.C.); diego.marques.lopes@gmail.com (D.M.L.); rsa_q@hotmail.com (R.S.A.); abcsimas@ippn.ufrj.br (A.B.C.S.); 4 Institute of Chemical, Biological, Radiological and Nuclear Defense (IDQBRN), Brazilian Army Technological Center (CTEx), Rio de Janeiro 23020-470, Brazil

**Keywords:** Wnt pathway, flavonoids, natural compounds, GSK3, memory deficit, neurodegenerative disease, aging, N2A cells

## Abstract

**Background**: The Wnt/β-catenin signaling pathway plays a pivotal role in embryonic development, maintenance of the central nervous system, and the formation of neuronal circuits. Disruption of this pathway is closely associated with oncogenesis and neurodegenerative diseases, notably Alzheimer’s disease. Flavonoids such as quercetin derivatives have emerged as promising neuroprotective agents. This study investigates the impact of 3-*O*-methylquercetin (3*O*MQ), a methylated quercetin metabolite, on Wnt/β-catenin signaling and its potential relevance in neurodegenerative disease models. **Methods**: The ability of 3*O*MQ to modulate Wnt/β-catenin activity was analyzed using a luciferase-based reporter assay in both neural and non-neural cell lines. Cell viability assays evaluated cytotoxicity at various concentrations. We mapped 3*O*MQ activity within the pathway using targeted cell signaling experiments. Docking and molecular dynamics simulations suggested glycogen synthase kinase 3β (GSK3β) as a putative target of 3*O*MQ. Finally, we employed a mouse model of acute amyloid-β oligomer (AβO) toxicity to assess the in vivo effects of 3*O*MQ on spatial memory and Wnt-related gene expression. **Results**: We compared the flavonoids quercitrin, quercetin, and 3-O-methylquercitrin (3*O*MQ) with pharmacologically active compounds in a gene reporter assay (TOPFLASH) using Wnt-sensitive RKO cells treated with Wnt3a-conditioned medium. XAV-939 and PNU-74654 showed inhibitory activity, while BIO, CHIR99021, quercitrin, and 3*O*MQ enhanced the Wnt/β-catenin pathway. Notably, 3*O*MQ potentiated this pathway at concentrations 5–10 times lower than quercitrin and outperformed 1 μM BIO at 10 μM without cytotoxicity, highlighting its remarkable potency. Mechanistically, 3*O*MQ acts downstream of initial membrane activation and upstream of the β-catenin destruction complex. Consistently, molecular docking indicates a strong interaction with GSK3, a central regulator of the pathway. In adult mice, 3*O*MQ administration prevented AβO-induced recognition memory deficits and favored normalization of Wnt-related gene expression. **Conclusions**: These findings identify 3*O*MQ as a potent positive modulator of the Wnt/β-catenin pathway, with both in vitro and in vivo neuroprotective effects. Targeting Wnt signaling with compounds such as 3*O*MQ holds promise for maintaining neuronal health and developing therapeutic strategies for neurodegenerative conditions.

## 1. Introduction

Aging primarily drives several diseases, including cancer and neurodegenerative disorders like Alzheimer’s and Parkinson’s [1,2,3,4]. Wnt signaling directly controls the proliferation and maintenance of neural stem cells (NSCs), thereby supporting neurogenesis and brain plasticity [5,6,7,8,9,10]. Studies demonstrate that strict regulation of this pathway prevents NSC loss and supports ongoing neurogenesis [5,11,12]. In adults, neurogenesis primarily occurs in the subventricular zone of the lateral ventricle and the subgranular zone of the hippocampal dentate gyrus; Wnt signaling in these regions orchestrates neuronal differentiation and cellular balance [13]. Overexpressing the Wnt3 ligand enhances neurogenesis in adult hippocampal progenitors, whereas blocking the pathway nearly abolishes this process in both in vivo and in vitro models [14].

Reduced Wnt signaling is linked to degeneration in several tissues, including the CNS, underscoring its role in protecting against age-related neuropathologies [9]. Activating this pathway can directly alleviate cognitive deficits in models, indicating that targeted Wnt signaling may help slow cognitive decline and combat neurodegenerative diseases [15,16]. With few effective treatments available, research on Wnt signaling holds strong potential for new therapeutic strategies [4,8]. Clarifying its regulation and methods for selective activation could reveal new avenues for maintaining neuronal integrity, supporting healthier aging, and enhancing the quality of life for older adults.

The Wnt signaling pathway is highly conserved in animals and is critical from embryogenesis onward. Its activation controls main processes such as proliferation, differentiation, polarity, and cell fate, making it essential for tissue balance [17,18]. Nineteen Wnt ligands, secreted glycoproteins, are regulated by auxiliary proteins influencing their stability and distribution [19,20]. The pathway has three branches: canonical (β-catenin-dependent), planar cell polarity (PCP), and Wnt/Ca^2+^, each with unique signaling roles [21,22]. The canonical pathway stabilizes β-catenin to regulate gene transcription, affecting stem cell renewal, cell cycle, and cell integrity [18,23]. Feedback mechanisms regulate receptor levels and β-catenin degradation, thereby maintaining a balance in signaling [24,25].

Wnt signaling is crucial for neurogenesis and neural plasticity in the central nervous system (CNS) throughout an individual’s lifespan. During development, the Wnt pathway plays a crucial role in body axis formation and neural patterning, regulating proliferation, neuronal differentiation, and synaptic connectivity [26,27,28,29]. Recent evidence highlights the importance of precise temporal regulation of Wnt signaling, demonstrating that the histone demethylase KDM5C acts as a key upstream regulator of Wnt/β-catenin signaling during a critical developmental window, ensuring the timely transition from primary to intermediate neural progenitors. Loss of KDM5C leads to premature Wnt activation, delayed neurogenesis, and long-term cognitive deficits, highlighting the importance of precise temporal control of Wnt signaling in neurodevelopment [30]. In adults, its activity is limited to neurogenic niches, such as the subgranular zone of the dentate gyrus and the subventricular zone, where it regulates the self-renewal and differentiation of neural stem cells (NSCs) [14,31]. Recent evidence also demonstrates that Wnt signaling sustains adult hippocampal neurogenesis, with the NEIL3 protein acting through this pathway to maintain progenitor cell pools and support the separation of behavioral patterns, linking Wnt activity to cognitive performance [32]. Beyond neurogenesis, Wnt signaling also influences synaptic plasticity and the maintenance of excitatory synapses, which are crucial for memory and learning [33,34,35,36]. Dysregulation can lower neurogenesis and contribute to cognitive deficits in aging and neurodegenerative diseases, such as Alzheimer’s and Parkinson’s [37,38,39,40]. Lower Wnt ligand levels in the aging hippocampus correlate with fewer new neurons and worse memory [31,41].

Dysregulation of the Wnt pathway is strongly linked to neurodegenerative diseases. Research shows that Wnt pathway inhibition during brain aging and AD lowers nuclear β-catenin, compromising neuron survival and synaptic maintenance [1,42]. Increased levels of inhibitory components, such as Dickkopf-1 (Dkk1), in AD models further block Wnt signaling and exacerbate cognitive impairment [43,44]. Animal models show that activating the Wnt pathway can reverse cognitive deficits and protect against β-amyloid-induced neurotoxicity [16]. In PD, dopaminergic neuron loss is associated with negative Wnt regulation, and studies suggest that restoring its activity may have therapeutic benefits [45,46]. These findings highlight the potential of modulating Wnt signaling to slow neurodegeneration and develop neuroprotective therapies.

Flavonoids are polyphenolic plant compounds with antioxidant, anti-inflammatory, and neuroprotective properties [47,48,49,50]. Flavonoids modulate inflammation and protect cells from oxidative damage, both key factors in neurodegeneration. In neurodegenerative disease models, flavonoids lower pro-inflammatory cytokines, such as TNF-α, IL-6, and COX-2, while increasing neurotrophic and anti-inflammatory factors, including IL-10 and BDNF, thereby supporting neuronal homeostasis [51,52,53,54]. Recent research has shown that flavonoids, such as baicalein, kaempferol, isorhamnetin, and quercitrin, can stabilize β-catenin through the Wnt/β-catenin pathway, potentially preventing neurodegeneration [51,55,56,57]. These attributes suggest therapeutic promise for neurodegenerative diseases, as Wnt activation enhances synaptic plasticity and neurogenesis in models of brain aging and neurodegeneration [11,16,37,58,59]. However, most of the Wnt pathway modulators described to date are inhibitors, and the number of natural activators, such as flavonoids, is still limited, highlighting the need to identify new compounds for positive modulation of the pathway [60,61,62].

This study was undertaken to explore the flavonol 3-*O*-methylquercetin (3*O*MQ), which was identified as a positive modulator of the canonical Wnt pathway in a screen compared to other Wnt-modulator compounds tested using a Wnt-specific gene reporter assay. To validate pathway responsiveness and establish reference controls, well-characterized Wnt modulators were included in the assays: CHIR99021 and BIO as canonical Wnt activators through GSK3β inhibition, and PNU-74654 as a β-catenin/TCF interaction inhibitor. Our data show that 3*O*MQ enhanced the Wnt/β-catenin signaling pathway at an optimal concentration of 10 μM. 3*O*MQ also decreased the viability of neural and non-neural cell lines, with effects becoming noticeable at concentrations of 30 μM after 48 h. Cell signaling experiments showed that 3*O*MQ acts specifically, functioning downstream of membrane activation and upstream of the β-catenin destruction complex. Docking and molecular dynamics simulations revealed stable interactions between 3*O*MQ and GSK3β, suggesting this kinase as a potential target of the flavonoid in the Wnt/β-catenin signaling pathway. To address the function of 3*O*MQ, we evaluated its efficacy in preventing memory deficits induced by the central neurotoxins involved in Alzheimer’s Disease—Amyloid-β oligomers (AβO) in a murine model. Altogether, our data indicate that 3*O*MQ potentiates Wnt/β-catenin signaling, likely by inhibiting GSK activity, and is a potential compound for reducing memory deficits. Understanding the mode of action of 3*O*MQ on Wnt/β-catenin signaling could guide future therapies for neuroprotection and neurodegenerative disorders.

## 2. Results

### 2.1. The Flavonol 3-O-Methylquercetin (3OMQ) Potentiates Wnt/β-Catenin

**Figure 1:**
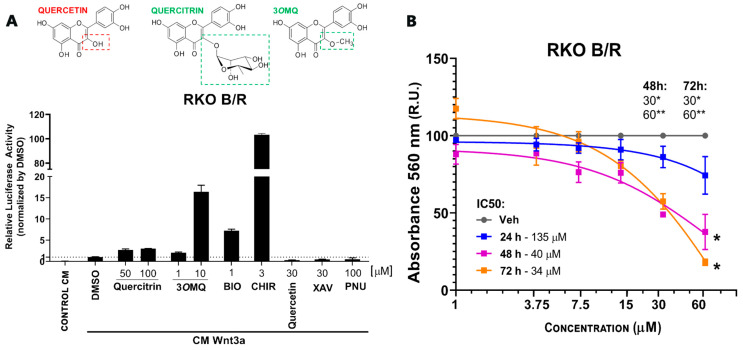
A Wnt/β-catenin specific reporter in RKO cells reveals a potential Wnt signaling modulator. (**A**) Reporter gene assay (TOPFLASH) in RKO cells treated with Wnt3a conditioned medium (Wnt3a CM) in the presence of potentiating molecules Quercitrin (50 and 100 µM), 3*O*MQ (1 and 10 µM), BIO (1 µM), or inhibitory molecules Quercetin (30 µM), PNU-74654 (100 µM), and XAV939 (30 µM), used as reference modulators. (**B**) Cell viability assay (MTT) in RKO cells treated for 24–72 h with increasing concentrations of 3*O*MQ (1, 3,75, 7,5, 15, 30, and 60 µM). Graphs represent mean ± standard deviation (SD). Statistical analysis was performed using one-way ANOVA followed by Tukey’s test. * *p* < 0.01; ** *p* < 0.0001.

Previous studies by our group have shown that the flavonoids quercitrin and isoquercitrin [63,64] inhibit the Wnt/β-catenin pathway both in vivo and in vitro, preventing tumor growth in colorectal cancer cells. We have also demonstrated that the flavonol quercitrin enhances the Wnt/β-catenin pathway through GSK3 inhibition and exhibits synaptogenic activity [56]. These findings suggest that the chemical structure of flavonoids confers distinct properties in modulating the Wnt/β-catenin signaling pathway. To identify Wnt/β-catenin pathway potentiators with activity similar to that of commercial activators, we compared the flavonoids quercitrin, quercetin, and 3-O-methylquercitrin with commercial compounds known to modulate this pathway pharmacologically. We used the gene reporter assay (TOPFLASH) in Wnt-sensitive RKO cells treated with Wnt3a-conditioned medium from L cells (L-Wnt3a CM). XAV-939, PNU-74654 (2-Phenoxybenzoic acid-[(5-methyl-2-furanyl)methylene]hydrazide), and quercetin showed inhibitory activity, while BIO, CHIR99021, quercitrin, and 3*O*MQ enhanced the effects induced by L-Wnt3a CM. Surprisingly, 3*O*MQ potentiated the Wnt/β-catenin pathway at concentrations 5–10 times lower than quercitrin, and at 10 μM, it outperformed 1 μM BIO (Figure 1A). Next, we assessed the cytotoxic effects of 3*O*MQ on RKO cells using a cell viability assay at concentrations ranging from 1 to 60 μM over 24, 48, and 72 h. Our results exclude cytotoxic effects of 3*O*MQ at all concentrations tested over 24 h, but indicate significant effects on cell viability at concentrations above 30 μM after 48 h (Figure 1B). Overall, these findings demonstrate that 3*O*MQ potentiates the Wnt/β-catenin pathway within the 1–10 μM range without substantially impacting RKO cell viability.

### 2.2. 3OMQ Potentiates Wnt3a Activity and Accumulates Nuclear Beta-Catenin in Neuro-2A Cells

**Figure 2:**
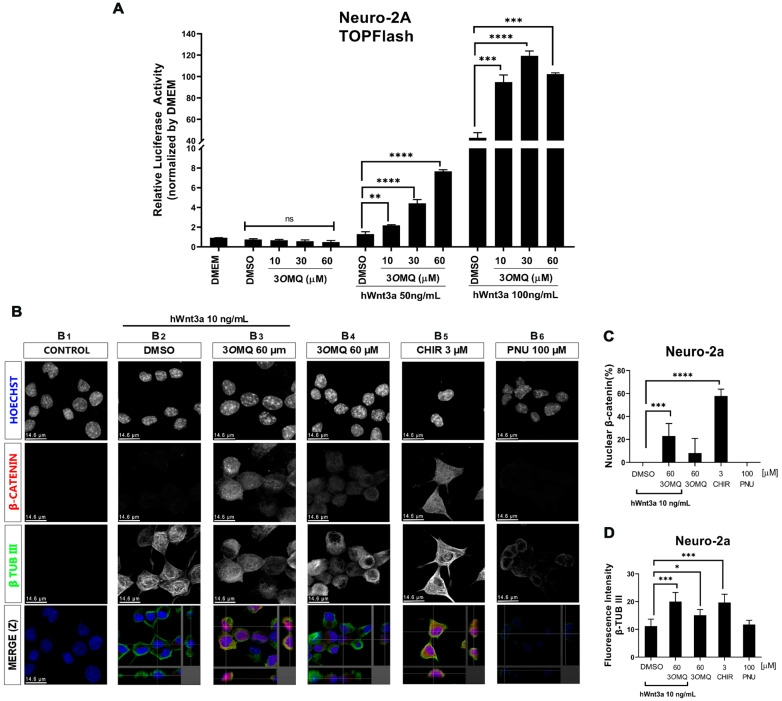
3*O*MQ potentiates the canonical Wnt pathway in the presence of Wnt3a ligand in Neuro-2A cells. (**A**) Reporter gene assay (TOPFLASH) in Neuro-2a lineage cells treated with 10, 30, and 60 µM 3*O*MQ and co-treated with 50 or 100 ng/mL of hWnt3a recombinant protein. (**B**) Indirect immunofluorescence assay in Neuro-2a lineage cells treated with 60 µM 3*O*MQ with or without hWnt3a recombinant protein (10 ng/mL), 3 µM CHIR, and 100 µM PNU. Immunofluorescence for early neuronal cell differentiation marker β-TUBIII (green), nuclei stained with Hoechst (blue) and β-catenin (red), showing that 3*O*MQ increases nuclear translocation of β-catenin in Neuro-2a cells after 24 h of treatment. (**C**) Quantification of the count ratio of nuclear β-catenin-positive cells. (**D**) Quantification of fluorescence intensity for β-TUB III positive cells. Scale bar represents 14.6 μm. Statistical significance was set as follows: *p* < 0.01 (*), *p* < 0.005 (**), *p* < 0.001 (***), *p* < 0.0001 (****), and ns = not significant. Graphs represent mean ± SD.

We tested the effect of 3*O*MQ on the Wnt/β-catenin pathway in Neuro-2A cells. For this, we used a TOPFLASH reporter gene assay specific for the Wnt/β-catenin pathway in the presence and absence of recombinant Wnt3a ligand protein. At concentrations of 10, 30, and 60 μM, 3*O*MQ did not activate the reporter gene in Neuro-2a cells in the absence of Wnt3a ligand (Figure 2A). However, with 50 and 100 ng/mL Wnt3a, 3*O*MQ significantly enhanced the activity of the TOPFLASH reporter gene in a concentration-dependent manner. The presence of nuclear β-catenin indicates activation of the canonical Wnt pathway. Therefore, we evaluated nuclear β-catenin in Neuro-2A cells using immunofluorescence and confocal imaging. Untreated or 10 ng of Wnt3a-treated Neuro-2A cells showed no reactivity for nuclear beta-catenin and were identified with an anti-β-tubulin III antibody, confirming their neural identity (Figure 2B–D). Consistent with previous results, 60 μM of 3*O*MQ combined with 10 ng of Wnt3a showed significant immunostaining for nuclear beta-catenin, similar to the effect seen with the Wnt/β-catenin pathway activator CHIR at 3 μM (Figure 2B–D). We observed that 3*O*MQ induced residual levels of nuclear beta-catenin in Neuro-2A cells, even in the absence of Wnt3a. As expected, neuro-2A cells treated with the Wnt/β-catenin pathway inhibitor PNU did not show nuclear β-catenin labeling (Figure 2B–D). Overall, these results demonstrate that Neuro-2A cells respond to the Wnt/β-catenin pathway and that 3*O*MQ can enhance the activity of a specific reporter gene for the Wnt/β-catenin pathway, promoting nuclear β-catenin accumulation.

### 2.3. 3OMQ Induces Cytotoxic Effects on Neural Cell Lineages

**Figure 3:**
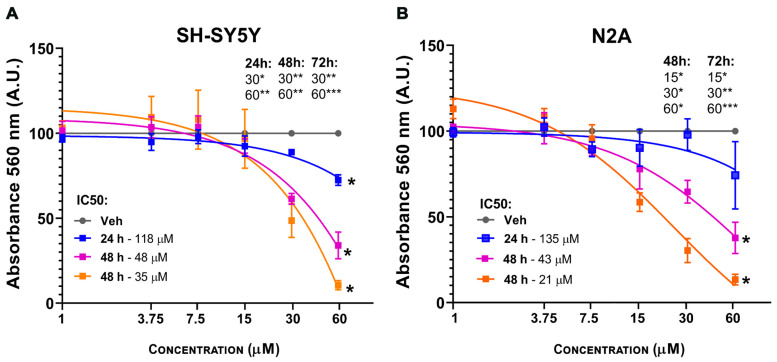
3*O*MQ induces cytotoxic effects on neural cell lineages. (**A**,**B**) Cell viability assay (MTT) in SH-SY5Y and Neuro-2a cell lines treated for 24 h with 3*O*MQ at 1, 3.75, 7.5, 15, 30, and 60 μM. Graphs represent mean ± standard deviation (SD). Statistical significance was set as follows: *p* < 0.01 (*), *p* < 0.005 (**), *p* < 0.001 (***). Graphs represent mean ± SD.

Two neuronal cell lines (Neuro-2a and SH-SY5Y) were evaluated for viability using the MTT assay at 3*O*MQ concentrations ranging from 1 to 60 μM. SH-SY5Y cells, a widely used human cell line derived from neuroblastoma, exhibited toxicity at concentrations above 30 μM across 24, 48, and 72 h, with an IC50 of 35 μM at 72 h (Figure 3A). In contrast, Neuro-2a (N2A) cells, an immortalized mouse neuroblastoma cell line, showed signs of toxicity only after 48 h, starting at 15 μM, with an IC50 of 21 μM at 72 h (Figure 3B). These findings confirm that 3*O*MQ does not exert cytotoxic effects at concentrations between 1 and 10 μM, consistent with previous observations using RKO cells. Overall, the results indicate that neuronal cells are more sensitive to 3*O*MQ than rat-derived non-neuronal cell lines, while still maintaining a non-toxic profile at lower concentrations.

### 2.4. 3OMQ Does Not Activate the Wnt/β-Catenin Pathway at the Destruction Complex Level When GSK3 Is Inhibited

**Figure 4:**
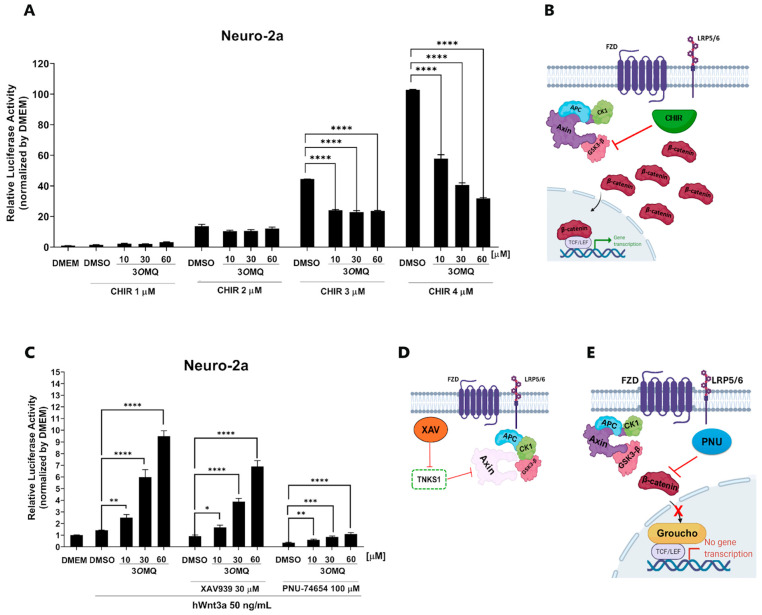
3*O*MQ does not activate the Wnt/β-catenin pathway at the destruction complex level when GSK3 is inhibited. (**A**) Reporter gene assay (TOPFLASH) performed in Neuro-2a cells treated with 3*O*MQ at 10, 30, and 60 µM, co-treated with the GSK3 inhibitor CHIR at 1, 2, 3, and 4 µM. Graphs represent mean ± standard deviation (SD). (**B**) Schematic representation of the mechanism of action of CHIR99021, which inhibits GSK3-β, leading to the disassembly of the β-catenin destruction complex. This inhibition results in β-catenin accumulation in the cytoplasm and its translocation into the nucleus, thereby activating the canonical Wnt pathway. (**C**) 3*O*MQ reverses inhibition of the Wnt/β-catenin pathway at the level of destruction complex, reporter gene assay (TOPFLASH) in Neuro-2a lineage cells treated with 3*O*MQ at 10, 30 and 60 µM with hWnt3a recombinant protein (50 ng/mL), co-treated with 30 µM XAV939 or 100 µM PNU-74654. (**D**) Mechanism of action of XAV939, a tankyrase inhibitor. Tankyrase normally promotes the degradation of AXIN, a key component of the β-catenin destruction complex. By inhibiting tankyrase, XAV939 stabilizes AXIN, reinforcing the destruction complex and inhibiting Wnt signaling. (**E**) Representation of the mechanism of action of PNU-74654, which inhibits the canonical Wnt pathway by binding to β-catenin. This binding prevents β-catenin from interacting with TCF/LEF transcription factors, thereby reducing the transcription of Wnt target genes. (**B**,**D**,**E**) Created in BioRender. Bonfim, D. (2025), https://BioRender.com/cbihnn3 (accessed on 2 November 2025). Statistical significance was set as follows: *p* < 0.01 (*), *p* < 0.005 (**), *p* < 0.001 (***), *p* < 0.0001 (****). Graphs represent mean ± SD.

To elucidate the possible mechanism of action of 3*O*MQ, we performed a TOPFLASH reporter assay to assess Wnt/β-catenin pathway activation in Neuro-2A cells treated with various concentrations of 3*O*MQ (1, 10, and 60 μM; Figure 4A). We also included the GSK3 inhibitor CHIR (1–4 μM) as a control, given its established role in promoting β-catenin accumulation by blocking its phosphorylation and degradation (Figure 4B). Our findings reveal that 3*O*MQ’s ability to activate the pathway is significantly reduced in the presence of CHIR, particularly at higher CHIR concentrations, suggesting a competition between the two compounds. This observation suggests a potential interplay between 3*O*MQ’s mechanism and GSK3 activity, implying that 3*O*MQ may act upstream or at the level of GSK3 to modulate Wnt signaling. To further dissect the site of action of 3*O*MQ within the pathway, we examined its effects in combination with two other Wnt pathway inhibitors: XAV939 (30 μM), which stabilizes Axin and promotes β-catenin degradation by inhibiting tankyrase (Figure 4D); and PNU-74654 (100 μM), which disrupts β-catenin’s interaction with T cell factor 4, an essential step for its nuclear transcriptional activity (Figure 4E). Interestingly, while co-treatment with XAV939 did not significantly alter 3*O*MQ activity, co-treatment with PNU-74654 effectively suppressed it (Figure 4C). Conceptually, these results suggest that 3*O*MQ’s action is independent of Axin-mediated β-catenin degradation, as modulating this component did not affect its activity. In contrast, the sensitivity of 3*O*MQ’s effect to PNU-74654 indicates that 3*O*MQ acts prior to or involves the step of β-catenin’s nuclear translocation and its subsequent modulation of gene transcription. Overall, these data point to a mechanism for 3*O*MQ that is likely acting downstream to Axin stabilization but does not work in synergy with the GSK3 inhibitor CHIR.

### 2.5. Molecular Modeling Highlight GSK3-β as the Possible Molecular Target of 3OMQ

**Figure 5:**
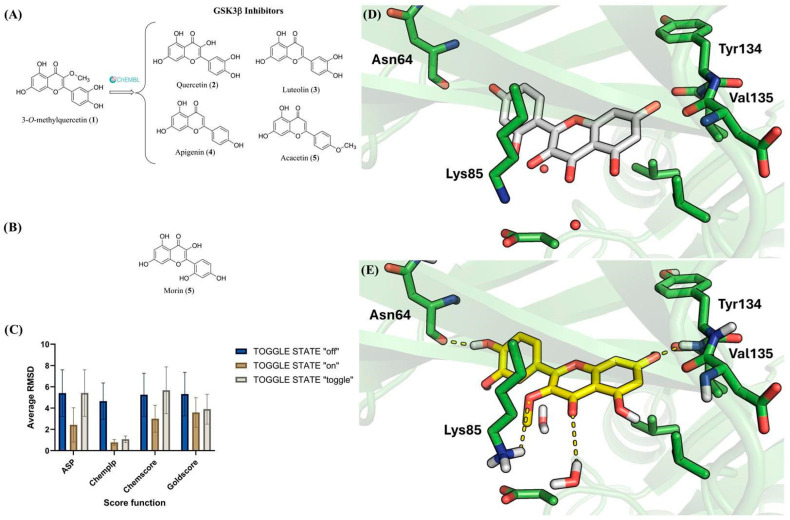
Chemical Structures of Flavonoid GSK3-β Inhibitors and docking analysis of 3*O*MQ. (**A**) Chemical structure of similar GSK3β inhibitors to 3*O*MQ found the ChEMBL database. (**B**) Chemical structure of morin (5), a flavonoid co-crystallized within the active site of GSK3-β. (**C**) Average RMSD obtained from redocking the co-crystallized ligand (6AE3). The data were calculated as the average RMSD values for the 10 solutions generated for each scoring function (ASP, CHEMPLP, CHEMCORE, GOLDSCORE) using three different water molecule settings (TOGGLE STATE: off; on; toggle). (**D**) The binding mode of morin (**6**) (carbons in white) in GSK3-β (PDB: 6AE3). (**E**) Analysis of the possible binding mode of 3*O*MQ (carbons in yellow) in GSK3-β (PDB: 6AE3) through molecular docking.

Structural similarity searches based on molecular fingerprints are valuable tools in ligand-based virtual screening strategies [65,66,67,68]. In these approaches, the higher the similarity between reference compounds and query molecules, the greater the likelihood that the latter will display activity against the same biological target. In the case of 3*O*MQ, a reverse similarity analysis was performed, comparing this compound with reference ligands for several protein targets (LRP5/6, Frizzled, Wnt3a, Wnt5a, GSK3-β, and Dvl) to identify the most plausible targets associated with Wnt pathway activation. This could provide a mechanistic explanation for the neuroprotective effects attributed to 3*O*MQ. Among the evaluated proteins, GSK3-β was the only target for which reported ligands exhibited ≥50% similarity to 3*O*MQ according to Tanimoto coefficients [69]. Figure 5A depicts representative GSK3-β inhibitors structurally similar to 3*O*MQ, with IC_50_ values in the low micromolar range. Notably, these inhibitors, like 3*O*MQ, belong to the flavonoid class of natural products. Taken together, these findings strongly suggest that 3*O*MQ may act as a GSK3-β inhibitor.

To investigate the potential interaction mode of 3*O*MQ with GSK3-β, molecular docking studies were performed using the crystal structure available in the Protein Data Bank under the PDB code 6AE3 [70]. This structure was selected due to the high structural similarity between the co-crystallized ligand, morin (**6**)—a flavonoid and natural regioisomer of quercetin (**5**) in the catechol ring (Figure 5B)—and 3*O*MQ. Although PDB 6AE3 corresponds to GSK3-β from *Mus musculus*, its use is appropriate for modeling human interactions, as the alignment of the primary sequences of GSK3-β from *Mus musculus* and *Homo sapiens*, using the Clustal Omega program in the UniProt database (https://www.uniprot.org/align, accessed on 2 November 2025), revealed 99.05% sequence identity.

Prior to docking 3*O*MQ into GSK3-β, the docking methodology was validated. A common validation strategy involves redocking the co-crystallized ligand and comparing the predicted and experimental binding modes. In this approach, root-mean-square deviation (RMSD) values are used as a measure of accuracy: the lower the RMSD, the closer the predicted pose is to the crystallographic reference. Typically, a methodology is considered reliable when RMSD values fall below the resolution of the crystallographic structure (Å) [71]. The crystal structure, with PDB code 6AE3 [70], has a resolution of 2.14 Å. An additional factor to consider in docking validation is the role of water molecules, which may participate in ligand recognition either by mediating hydrogen bonds between the ligand and protein or by being displaced, thereby providing entropic contributions to complex formation [72,73]. In PDB 6AE3, several water molecules are located near the co-crystallized ligand, highlighting the need to evaluate their influence on docking performance. The GOLD software (v. 2025.1.0) allows three configurations for water treatment, which is called TOGGLE STATE: “off” (water molecule excluded), “on” (water molecule retained), and “toggle” (GOLD determines whether the water molecule is retained or displaced by the ligand). Furthermore, GOLD provides four scoring functions (ASP, CHEMPLP, CHEMSCORE, and GOLDSCORE) to rank docking poses generated by its genetic algorithm. Each scoring function was tested in redocking experiments under the three water configurations described above. By default, GOLD generates 10 final poses per ligand, resulting in 120 solutions in total (10 poses × 4 scoring functions × 3 water configurations). For each pose, RMSD values were calculated, and the average RMSD values for each scoring function under the three water treatments are presented in Figure 5C. Analysis of Figure 5C shows that only the CHEMPLP scoring function, when configured with TOGGLE STATE set to “on” or “toggle”, successfully reproduced the experimental binding mode of the co-crystallized ligand. These configurations were therefore selected as the most appropriate for investigating the interactions of 3*O*MQ with GSK3-β (PDB: 6AE3). Docking of 3*O*MQ was subsequently performed using the TOGGLE STATE: “toggle” option.

The binding mode of the co-crystallized ligand and the predicted binding pose of 3*O*MQ are shown in Figure 5D,E, respectively. Similarly to the co-crystallized ligand, 3*O*MQ engages in hydrogen bonding with the backbone of Val135 in the hinge region of GSK3-β—an interaction known to be critical for kinase inhibitor recognition at the ATP-binding site. Additionally, 3*O*MQ establishes hydrogen bonds with a water molecule in the ATP-binding site, the side chain of Lys85, and the backbone of Asn64. Notably, the docking score obtained for 3*O*MQ (score = 57) is comparable to that of the co-crystallized ligand (score = 59). Taken together, these results reinforce the hypothesis that 3*O*MQ can act as a GSK3-β inhibitor.

**Figure 6:**
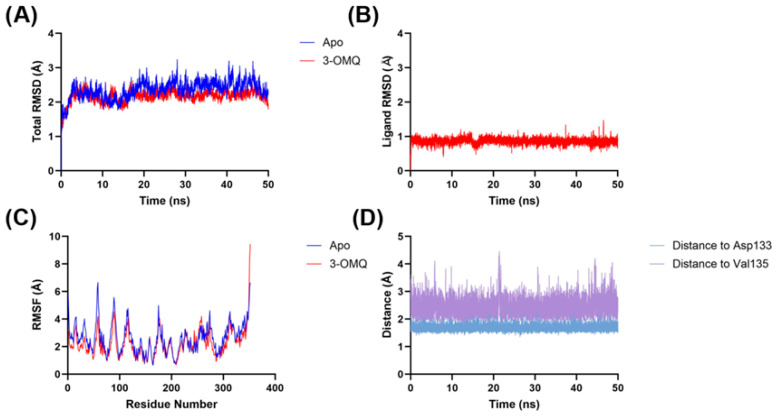
Molecular dynamics analyses of the 3*O*MQ–GSK3-β complex (PDB: 6AE3) calculated through molecular docking, compared to the apo form of GSK3-β (PDB: 6AE3). (**A**) total RMSD of apo form and the 3*O*MQ–GSK3-β complex. (**B**) 3*O*MQ RMSD. (**C**) RMSF of apo form and the 3*O*MQ–GSK3-β complex. (**D**) Hydrogen bond distance of 3*O*MQ in relation to Asp133 and Val135 of GSK3-β.

The molecular dynamics analysis of the GSK3β–3*O*MQ complex demonstrated significant structural stability throughout the simulation (Figure 6). Comparison between the apo form of the protein and the ligand–protein complex showed that the overall RMSD remained between 2 and 3 Å in both cases, indicating that ligand binding did not induce major conformational changes in the protein. Consistently, the RMSF profiles were also largely superimposable, suggesting that the residual flexibility of GSK3β is essentially unaffected by 3*O*MQ binding. The RMSD of the ligand alone remained below 1 Å, indicating that 3*O*MQ maintains a relatively stable position in the active site and confirming the reliability of the docking pose. Detailed inspection of interactions revealed two persistent hydrogen bonds throughout the simulation: one with Asp133 (~1.9 Å), which was not predicted by docking despite the ligand being in close proximity, highlighting the ability of molecular dynamics to capture subtle stabilizing interactions not evident in static models; and another with Val135 (2–3 Å), which corroborates an interaction suggested by docking, supporting the validity of the initial pose. Overall, these results indicate that 3*O*MQ is stably accommodated in the GSK3-β active site, maintaining key interactions that may contribute to ligand affinity and selectivity. The stability of the complex and the persistence of hydrogen bonds suggest that 3*O*MQ is a promising candidate for modulating GSK3β activity, providing valuable insights for future chemical optimization.

### 2.6. 3OMQ Prevents Recognition Memory Deficits Caused by AβO in a Murine Behavior Model

**Figure 7:**
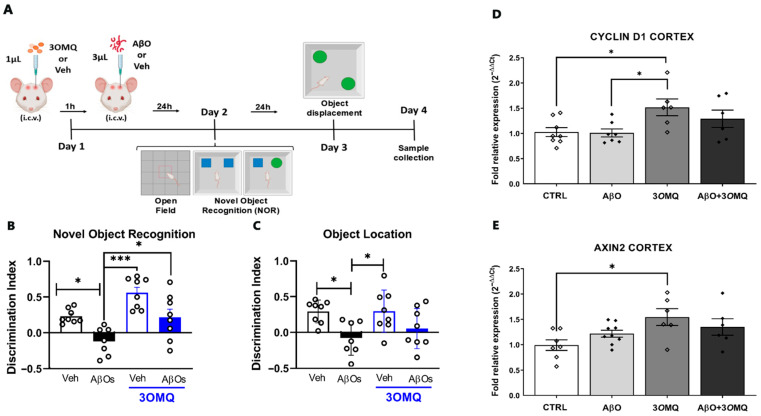
3*O*MQ prevents spatial memory deficits caused by AβO in a murine behavior model. (**A**) Experimental design of the behavioral assays. Female Swiss mice at postnatal day 60 (P60) received 1 μL of 3*O*MQ (10 μM working solution; 10 pmol/mice) or Vehicle (DMSO) via i.c.v., followed 1 h later by administration of 3 μL of Aβ (10 pmol/mice) or Vehicle (PBS). On Day 2, animals were subjected to the open field test and subsequently to the novel object recognition (NOR) test. On Day 3, the object location test was performed. Sample collection occurred on Day 4. Figures were created using BioRender (available at, https://BioRender.com/wjijlde accessed on 2 November 2025). (**B**) Exploration time of the novel object versus the familiar object in the novel object recognition test (*n* = 8 Veh, 7 AβOs, 8 Veh + 3*O*MQ, 8 AβOs + 3*O*MQ). (**C**) Exploration time of the displaced object versus the fixed object in the object location test (*n* = 8 Veh, 7 AβOs, 8 Veh + 3*O*MQ, 8 AβOs + 3*O*MQ). (**D**,**E**) Relative mRNA expression of Cyclin D1 (D, *n* = 7 Veh, 9 AβOs, 6 Veh + 3*O*MQ, 6 AβOs + 3*O*MQ) and Axin2 (E, *n* = 8 Veh, 7 AβOs, 6 Veh + 3*O*MQ, 6 AβOs + 3*O*MQ) genes in the cortex of animals treated with vehicle (Veh), β-amyloid oligomers (AβO), 3*O*MQ, or the combination AβO + 3*O*MQ. Data from behavioral tests are expressed as mean ± standard error of the mean (SEM), while PCR data are expressed as mean ± standard deviation (2^−ΔΔCt^) after normalization with reference genes. Statistical analysis was performed by one-way ANOVA followed by Tukey’s post-test. * *p* < 0.05; *** *p* < 0.0001.

Next, we investigated whether 3*O*MQ could prevent memory impairment induced by the central neurotoxins in Alzheimer’s disease, the oligomeric forms of amyloid-β peptide. To this, we used a mouse model of acute amyloid toxicity, widely used by our group and others [74,75], in which AβOs are injected into the lateral ventricles of adult mice, inducing several key features of the disease, including oxidative stress, synapse loss, tau aggregation, and cognitive deficit [43,44,74,75] (Figure 7A). In agreement with our previous findings, we observed that a single injection of AβOs resulted in a significant decrease in the discrimination index in the Novel Object Recognition test (NOR; Figure 7B, black bar) compared to vehicle-treated mice (white bar). Importantly, injection of 3*O*MQ prevented the AβO-induced cognitive impairment (Figure 7B, blue bar), while 3*O*MQ alone had no impact on memory formation in this task. Animals were also trained in a variation in the NOR test, which involves spatial information in the formed cognitive engram. In the object displacement test (Figure 7C), AβO-injected animals showed impaired performance when compared to Veh-injected mice, as shown by the significant decrease in exploration index in the test session (Figure 7C). However, treatment with 3*O*MQ was unable to prevent cognitive deficit induced by AβOs. This suggests that the effects of 3*O*MQ may be more limited to specific types of declarative entorhinal and perirhinal cortex-dependent memories, rather than spatial hippocampal-dependent memories. It is important to note that locomotor and exploratory behaviors were not affected by the treatment (Supplementary Figure S2), and that 3*O*MQ alone did not cause cognitive impairment or any signs of toxicity.

Molecular analysis of cortical tissue provided further insight into the effects of 3*O*MQ. Expression of Cyclin D1, a regulator of cell cycle and proliferation, was markedly elevated in the 3*O*MQ-treated group compared to both AβO-injected and control groups (Figure 7D), suggesting a pro-proliferative or pro-survival effect of the compound. Similarly, Axin2, a canonical target of the Wnt/β-catenin signaling pathway, was significantly increased in animals treated with 3*O*MQ relative to control (Figure 7E), indicating activation of this pathway. Notably, the combination of AβOs and 3*O*MQ failed to restore Cyclin D1 and Axin2 expression to the levels observed in the 3*O*MQ-only group, highlighting that AβO exposure partially impairs cortical Wnt signaling even in the presence of 3*O*MQ.

## 3. Discussion

This study strengthens the growing body of evidence that flavonoid derivatives can modulate the Wnt/β-catenin signaling pathway, a central regulator of neurodevelopment, plasticity, and neuroprotection [41,59]. Our investigation focused on 3-*O*-methylquercetin (3*O*MQ), a methylated quercetin derivative, analyzing its direct effects on canonical Wnt signaling, cell viability, potential molecular targets, and neuroprotective properties in a murine model.

Previously, we identified quercitrin as a flavonoid capable of inhibiting GSK3 and potentiating the Wnt/β-catenin pathway in vitro [56]. Building upon these findings, the present study evaluates 3*O*MQ, representing a logical progression in understanding how chemical modifications to the quercetin scaffold might impact Wnt pathway modulation. While quercitrin was characterized by glycosylation at position 3, 3*O*MQ contains a methyl group at this site, potentially altering bioactivity, cellular uptake, and metabolic stability.

Unlike more potent canonical activators, 3*O*MQ does not independently trigger Wnt/β-catenin signaling in a strong manner. Instead, it acts mainly as a potentiator, enhancing the response to Wnt ligands in a moderate and concentration-dependent way, as demonstrated by reporter gene and protein analyses. Specifically, 3*O*MQ was able to induce TOPFLASH reporter activity and increase nuclear β-catenin, but did not surpass the effects observed with benchmark activators such as BIO or CHIR99021. Interestingly, co-treatment experiments showed that when GSK3 was pharmacologically inhibited, 3*O*MQ did not further enhance pathway activation, supporting the interpretation that its mechanism of action is upstream or at the level of GSK3, in line with the hypothesis and previous quercitrin work.

Our molecular modeling and similarity search approach identified GSK3β as the most plausible target for 3*O*MQ, aligning with both our previous observations with quercitrin [56] and the other literature reporting flavonoids as kinase modulators [55]. Docking analysis indicated that 3*O*MQ fits well into the ATP-binding site of GSK3-β, establishing hydrogen bonds at the hinge region, typical of type 1, type 1.5, or type 2 kinase inhibitors [76]. However, docking inherently has limitations, as only the polar hydrogens of the protein are treated as flexible; water molecule treatment remains a critical constraint, and scoring functions show poor correlation with experimental affinities. To overcome these issues, molecular dynamics simulations were employed, confirming the stability of the complex over time and reinforcing that hinge interactions observed for 3*O*MQ are not artifacts of the docking procedure [77].

A significant extension from cellular assays to in vivo application was achieved through behavioral testing in a murine model of acute amyloid-β oligomer (AβO) neurotoxicity. Indeed, the Wnt/β-catenin signaling pathway has long been implicated in the pathophysiology of Alzheimer’s Disease [78], and activation of this pathway has shown promising effects in several models of the disease [16,59]. Here, we found that 3*O*MQ prevented spatial memory impairment induced by AβO and normalized the expression of Wnt target genes in cortical tissue. These results suggest that 3*O*MQ, like quercitrin before it, may have therapeutic potential in counteracting cognitive deficits associated with impaired Wnt signaling [59]. Importantly, the lack of toxicity and reversal of AβO-induced cognitive impairment seen in mice treated with 3*O*MQ suggests these compounds are promising for treatment of neurodegenerative conditions such as Alzheimer’s disease, although additional studies should be performed to assess blood–brain barrier permeability, toxicity to different organs and long-term safety profile, among others. Moreover, additional preclinical studies should address the potential of 3*O*MQ in chronic models of AD, including transgenic mice models, to address the impact of this compound on other landmark features of the disease, including synapse loss, tau phosphorylation and aggregation, Aβ accumulation, and neuronal death. A recent study indicated that endogenous lithium deficiency leads to increased activation and expression of GSK3β. Furthermore, treatment with CHIR was found to reverse the elevated deposition of amyloid-beta (Aβ) associated with this deficiency [79]. In addition to lithium and CHIR, 3*O*MQ is emerging as a potential inhibitor of GSK3β. These compounds may collectively reactivate the Wnt/β-catenin signaling pathway in aged or pathological brain tissues.

Our data demonstrate that 3*O*MQ, within the tested concentration range, does not compromise cell viability, supporting a favorable safety profile comparable to that of other natural flavonoids [47]. This is an important aspect for pharmaceutical consideration, as Wnt pathway modulators face the ongoing challenge of maintaining on-target efficacy without unwanted side effects, particularly oncogenic risk [55].

Pharmaceutical development for CNS disorders requires compounds with reliable target engagement, acceptable toxicity, and brain availability. The progression from quercitrin to 3*O*MQ illustrates the classic medicinal chemistry paradigm—modification of a known bioactive scaffold to potentially enhance desired features (e.g., blood–brain barrier permeability via methylation) and further balance potency with safety. However, given that 3*O*MQ did not exceed the potency of established activators, its value may be most pronounced as a lead for scaffold optimization or rational design to increase GSK3 selectivity or enhance CNS pharmacokinetics. Therefore, this study should further explore 3*O*MQ’s interaction with GSK3 and upstream Wnt receptors, applying mutagenesis, kinase panel screens, and high-resolution structural analyses. Pharmacokinetic profiling may help improve central activity and specificity, possibly by using compounds that display similar or improved modulation at safer and lower dosages. As for the biological effects, this study would benefit from Long-term and Disease-Progression Models to determine 3*O*MQ’s capacity to preserve synaptic architecture, neurogenesis, and behavioral function over time.

## 4. Materials and Methods

### 4.1. Reagents and Cell Culture

Neuro-2A cells were kindly provided by Professor Julia Clarke (Instituto de Ciências Biomédicas, UFRJ). RKO pBAR/Renilla (B/R) and HEK293T cells were a generous gift from Professor Xi He (Boston Children’s Hospital, Harvard Medical School). L-cells and L-Wnt3a cell lines were obtained from ATCC, and conditioned medium was prepared according to the ATCC protocol. All cell lines were maintained in DMEM/F12 medium (Gibco, Waltham, MA, USA) supplemented with 10% fetal bovine serum (Gibco, Waltham, MA, USA) and incubated at 37 °C with 5% CO_2_ (Sanyo CO_2_ incubator). The reagents XAV939, PNU-74654, and CHIR99021 (Sigma, Burlington, MA, USA) were solubilized in DMSO (Sigma, Burlington, MA, USA) at 10 mM and stored at 20 °C. The flavonol 3-*O*-methylquercetin (3*O*MQ) was synthesized in partnership with Professor Alessandro Simas, by the Roderick A. Barnes Laboratory, of the Institute of Natural Products Research (IPPN). 3*O*MQ was solubilized in DMSO (Sigma, MO, USA) to a stock concentration of 50 mM and stored at −20 °C. 3-*O*-methylquercetin (3-methoxyquercetin) was synthesized either from the accessible flavonoids rutin or quercetin via a combination of the adapted literature procedures of selective OH-protections [80,81] and 3-*O*-methylation of the tri-*O*-benzyl or di-*O*-benzylquercetin intermediates [82]. The 1H NMR spectrum of the synthetic 3*O*MQ samples (See Supplementary Figure S1) is consistent with the literature data.

### 4.2. Cell Viability Assay

Cell viability was assessed by MTT assay (Invitrogen (Carlsbad, CA, USA), #M6494). Cells were seeded at 6 × 10^3^ cells/well in 96-well plates (≈1.9 × 10^4^ cells/cm^2^) with DMEM/F-12 + 10% FBS. After 24 h, the medium was replaced with fresh medium containing 3*O*MQ under different treatment conditions for 24, 48, or 72 h. Controls received DMEM/F-12 + 0.12% DMSO. At each time point, wells were washed with PBS and incubated with MTT (0.5 mg/mL, 100 μL) for 2 h at 37 °C. Formazan crystals were solubilized in 100 μL DMSO, and absorbance was read at 560 nm (GloMax^®^). Cell viability was expressed as % relative to untreated controls. All conditions were tested in triplicate in each of three independent experiments.

### 4.3. Immunocytochemistry

Neuro-2A cells were seeded at a density of 5 × 10^4^ cells on coverslips (Knittel Glass (Braunschweig, Germany), no. VD11515Y1A.01) placed in 24-well plates. After treatment, cells were fixed with 4% paraformaldehyde in phosphate-buffered saline (PBS) and permeabilized with PBS containing 0.5% Triton X-100 (Sigma Aldrich Saint Louis, MO, USA), no. T8787) for 5 min, followed by PBS washes. Blocking was performed for 1 h in PBS supplemented with 5% normal goat serum (NGS, Sigma Aldrich, no. 01-6201). Cells were then incubated for 1 h at room temperature with primary antibodies diluted in PBS containing 1% bovine serum albumin (BSA): rabbit anti-β-catenin (Sigma Aldrich, 1:200) and mouse anti-βIII tubulin (Sigma Aldrich, 1:200). After PBS washes, cells were incubated for 1 h at room temperature with secondary antibodies diluted 1:500 in PBS with 1% BSA: Alexa Fluor 546-conjugated anti-rabbit IgG (Thermo Fisher Scientific (Waltham, MA, USA), n°. A-11035) and Alexa Fluor 488-conjugated anti-mouse IgG (Thermo Fisher Scientific, n°. A-11001). Nuclei were stained with Hoechst 33342 (Thermo Fisher Scientific, n°. 62249) at 1:2000 for 15 min. Coverslips were washed with PBS and mounted using ProLong™ Glass Antifade Mountant (Invitrogen, Thermo Fisher Scientific, n°. P36980). Images were acquired with a Leica TCS SPE confocal microscope and analyzed with FIJI (ImageJ, NIH, Bethesda, MD, USA; version 1.53c, https://imagej.net/software/fiji/, accessed on 4 August 2023). Confocal images were acquired with a 63× oil-immersion objective and digital zoom was applied, providing the spatial resolution required to accurately assess nuclear localization. For each experimental condition, six independent confocal fields were collected to cover the entire cell population within the coverslip. All visible cells in these fields were analyzed to ensure representative sampling, compensating for the absence of wide-field overview images, which are incompatible with the high magnification required for signal discrimination. A nucleus was classified as β-catenin-positive when Alexa Fluor 555 fluorescence clearly overlapped with the Hoechst nuclear signal (405 nm excitation) in Z-stack reconstructions, indicating unambiguous nuclear colocalization. Nuclei lacking β-catenin fluorescence within the nuclear compartment were scored as negative.

### 4.4. Dual-Luciferase Assay

To evaluate the effect of 3*O*MQ on canonical Wnt activity, Neuro-2A cells were transduced with a lentiviral Wnt/β-catenin reporter construct produced in HEK293T cells using plasmids obtained from Addgene: psPAX2 (#12260), pCMV-VSV-G (#8454), 7TFP (#24308), LeGo-Renilla-BLAST (#122276), and pHIV-ZsGreen (#18121). After puromycin selection, transduced Neuro-2A (TOPFlash) cells and RKO B/R cells (2 × 10^4^/well), which already stably express the reporter, were seeded in 96-well plates and treated for 24 h with 3*O*MQ at different concentrations, in the presence or absence of Wnt3a-conditioned medium. Conditioned medium from L cells and 0.3% DMSO served as controls. Firefly and Renilla luciferase activities were measured with the Dual Luciferase Reporter Assay System (Promega) in a GloMax^®^ reader, and the results were normalized by the Firefly/Renilla ratio. All conditions were tested in triplicate in three independent experiments.

### 4.5. RNA Extraction and qPCR

Total RNA was extracted with the ReliaPrep™ RNA Miniprep System (Promega, Madison, WI, USA) and quantified by NanoDrop. One microgram of RNA was reverse transcribed using the GoScript™ kit (Promega), and qPCR was performed with GoTaq^®^ Master Mix (Promega) on an Applied Biosystems 7500 system. Gene expression was analyzed using primers specific for Cyclin D1 (forward: 5′-GCGTACCCTGACCAATCT-3′; reverse: 5′-ATCTCCTTCTGCACGCACTT-3′) and Axin (forward: 5′-TAGGCGGAATGAAGATGGAC-3′; reverse: 5′-CTGGT CAC CAA CAA GGAGT-3′), to evaluate the activity of the canonical Wnt signaling pathway. Actin (forward: 5′-GGC ATA GAG GTC TTT ACG GAT G-3′; reverse: 5′-TCA CTA TCG GCA ATG AGC G-3′) was used as an endogenous control, and relative expression levels were calculated using the 2^−ΔΔCt^ method. All reactions were performed in triplicate in three independent experiments, and the values were expressed by normalization to actin expression levels.

### 4.6. In Vivo Experiments

Swiss female mice (8–10 weeks old) were obtained from our own breeding facilities (UFRJ) and housed under 12 h light/dark cycle, controlled temperature, and humidity, and had access to food and water *ad libitum*. Preparation of Aβ oligomers (AβOs) was performed as previously described by Fontana et al., 2021 [83]. Briefly, the synthetic human Aβ1–42 peptide (Abcam, ab120301; 1 mg) was solubilized in 1,1,1,3,3,3-hexafluoro-2-propanol (HFIP; Sigma-Aldrich; 270 μL) and incubated in a closed vial for 1 h at room temperature. The solution was aliquoted into four vials (~65 μL each) and left to evaporate overnight to form Aβ–HFIP films, which were stored at −80 °C until use. For oligomerization, films were dissolved in DMSO (50 μL) and diluted in ice-cold PBS (490 μL) to a final concentration of 100 μM. The solution was incubated for 24 h at 4 °C and subsequently centrifuged at 14,000× *g* for 10 min at 4 °C to remove insoluble aggregates. The supernatant containing soluble AβOs was collected, quantified with the Pierce BCA Protein Assay kit (Thermo Scientific), aliquoted, and stored at −80 °C until use. The preparation used in experiments had a final concentration of 64 μM. For intracerebroventricular (i.c.v.) injections, animals were anesthetized with isoflurane (2.5%) and infused with 3*O*MQ (1 µL of a 10 µM working solution, 10 pmol/mice; DMSO/PBS vehicle) or vehicle (DMSO/PBS) using a Hamilton syringe, as described by Clarke et al. (2015) [74]. After 1 h, the same animals received β-amyloid oligomers (AβO, 10 pmol in 3 µL) or vehicle. The needle was kept in place for 30 s to avoid reflux, and animals showing signs of reflux or hemorrhage were excluded. Animals were randomly assigned into one of the four experimental groups, and control pf possible confounders were not applied. Exploratory and memory performance were evaluated using the open field test, the novel object recognition (NOR) test [84], and the object displacement test. NOR test was performed 24h after AβO injection. Mice underwent a 5 min training session with two identical objects, followed 90 min later by a 5 min test session in which one object was replaced with a novel one. Exploration time was recorded, and an exploration index was calculated as (time exploring novel − time exploring familiar)/total exploration time. In the displacement test, performed 48 h after AβO injection, one object was relocated during the test session, and preference for the displaced object was measured. Sessions were video-recorded and analyzed by a blinded experimenter. Animals that explored less than 10 s one of the objects were excluded from further analysis. Finally, 48 h after compound injection, mice were anesthetized (ketamine/xylazine, 100/10 mg/kg i.p.) and brains were collected for molecular analyses. Each animal was considered an independent experimental subject. Sample size was based on previous studies using the same behavioral tasks [85]. All researcher primary involved with conducting or analyzing the experiment were blind to experimental condition. Behavioral data are expressed as means ± SEM and the identification of outliers was evaluated using the ROUT test. D’Agostino-Pearson normality test used to assess the Gaussian distribution of the data. Statistical analysis of behavioral data was performed using one-way ANOVA followed by Tukey’s post-test (GraphPad Prism version 7.0 for Windows, GraphPad Software, La Jolla, CA, USA).

### 4.7. Statistical Analysis

Statistical analysis of the data was performed using one-way ANOVA associated with Dunnett’s or Tukey’s multiple comparison test (GraphPad Prism version 7.0 for Windows, GraphPad Software, La Jolla, CA, USA). All experiments were repeated at least three times and performed in triplicate. All graphs represent mean ± standard deviation (SD), or mean ± standard error of the mean (SEM), as indicated in the legends. Complete data are available upon request.

### 4.8. Molecular Modeling Analyses

Potential targets of 3*O*MQ were investigated by structural similarity analysis with ligands of LRP5/6, Frizzled, Wnt3a, Wnt5a, GSK3-β, and Dvl. Ligands were retrieved from ChEMBL, and circular fingerprints (ECFP4) [86] were compared with that of 3*O*MQ to calculate Tanimoto similarity indices [69] using KNIME. Docking was carried out only for targets with ligands showing ≥ 50% similarity to 3*O*MQ. The interaction mode was investigated using the GSK3-β crystal structure (PDB: 6AE3; [70]. Docking was validated by redocking the co-crystallized ligand with GOLD, testing four scoring functions (CHEMPLP, GOLDSCORE, CHEMSCORE, ASP) under three water configurations (“on”, “off”, “toggle”), with SPIN STATE fixed to “spin.” For 3*O*MQ, CHEMPLP with TOGGLE STATE set to “toggle” was selected. The binding site was defined at X:30.9180, Y:8.1130, Z:–9.7370, including all atoms within 10 Å. Molecular dynamics (MD) simulations were performed in FlarePro+ (Ametek, Berwyn, PA, USA) (OpenMM). The docked 3*O*MQ–GSK3-β complex was used as the starting structure, with truncated regions rebuilt via Loop Modeling. Ligands were minimized with OpenFF 2.2.0, proteins parameterized with AMBER, and systems solvated with the TIP3P water model. AM1-BCC charges were applied to ligands. After equilibration, 50 ns production runs were carried out, saving frames every 1 ps. Analyses included RMSD, RMSF, and monitoring of the distance between the ligand’s terminal benzyl group and hinge residues Asp133 and Val135 of GSK3-β.

## 5. Conclusions

This study extends the pharmacological mapping of flavonoid derivatives as Wnt pathway modulators from quercitrin to 3*O*MQ, confirming the latter’s moderate efficacy in canonical Wnt activation, its action at the level of GSK3, a reassuring safety profile, and neuroprotection in a disease-relevant model. The continuum from natural glycosides (quercitrin) to simpler methylated derivatives (3*O*MQ) charts a promising, stepwise path toward CNS drug candidates that may modulate neuroplasticity and resilience in neurodegenerative disease.

## Data Availability

The original contributions presented in this study are included in the article/Supplementary Material. Further inquiries can be directed to the corresponding author.

## Supplementary Materials

The following supporting information can be downloaded at https://www.mdpi.com/article/10.3390/ph18111680/s1. Figure S1: Representative image fields of the immunofluorescence assay used for nuclear b-catenin quantification; Figure S2: 1H NMR spectrum of 3*O*MQ; Figure S3: 3*O*MQ does not cause changes in locomotor or exploratory behaviors.

## Abbreviations

3*O*MQ	3-*O*-methylquercetin
Aβ	Amyloid beta
AβO(s)	Amyloid beta oligomer(s)
ANOVA	Analysis of Variance
BCA	Bicinchoninic Acid
BIO	6-bromoindirubin-3′-oxime
BSA	Bovine Serum Albumin
B/R	pBAR/Renilla cells
CM	Conditioned Medium
CNS	Central Nervous System
Ct	Cycle threshold
DAPI	4′,6-diamidino-2-phenylindole
DMSO	Dimethyl Sulfoxide
Dvl	Disheveled
ECFP4	Extended Connectivity Fingerprints, radius 4
FBS	Fetal Bovine Serum
FIJI	Fiji Is Just ImageJ
GSK3	Glycogen Synthase Kinase 3
GSK3-β	Glycogen Synthase Kinase 3 beta
HEK293T	Human Embryonic Kidney 293T
HFIP	Hexafluoro-2-propanol
HRP	Horseradish Peroxidase
ICV (i.c.v.)	intracerebroventricular
IgG	Immunoglobulin G
LRP5/6	Low-Density Lipoprotein Receptor-Related Protein 5/6
MD	Molecular Dynamics
MTT	3-(4,5-dimethylthiazol-2-yl)-2,5-diphenyltetrazolium bromide
NGS	Normal Goat Serum
NIH	National Institutes of Health
NOR	Novel Object Recognition
PBS	Phosphate-Buffered Saline
qPCR	Quantitative Polymerase Chain Reaction
RMSD	Root Mean Square Deviation
RMSF	Root Mean Square Fluctuation
SD	Standard Deviation
SEM	Standard Error of the Mean
SH-SY5Y	Human neuroblastoma cell line SH-SY5Y
TIP3P	Transferable Intermolecular Potential 3 Points
Veh	Vehicle
Wnt	Wingless/Integrated
WT	Wild type
