# Supplementary material for: Exploring the Impact of 3-O-Methylquercetin on Wnt/β-Catenin Pathway Activity and Its Potential in Neural Processes
Source: Pharmaceuticals (Basel). 2025 Nov 6;18(11):1680. doi: 10.3390/ph18111680 (PMC12655151; doi:10.3390/ph18111680)
Supplement: Supplementary file 1 [file pharmaceuticals-18-01680-s001.zip › pharmaceuticals-3939558-supplementary.pdf]

Supplementary Figure S1.

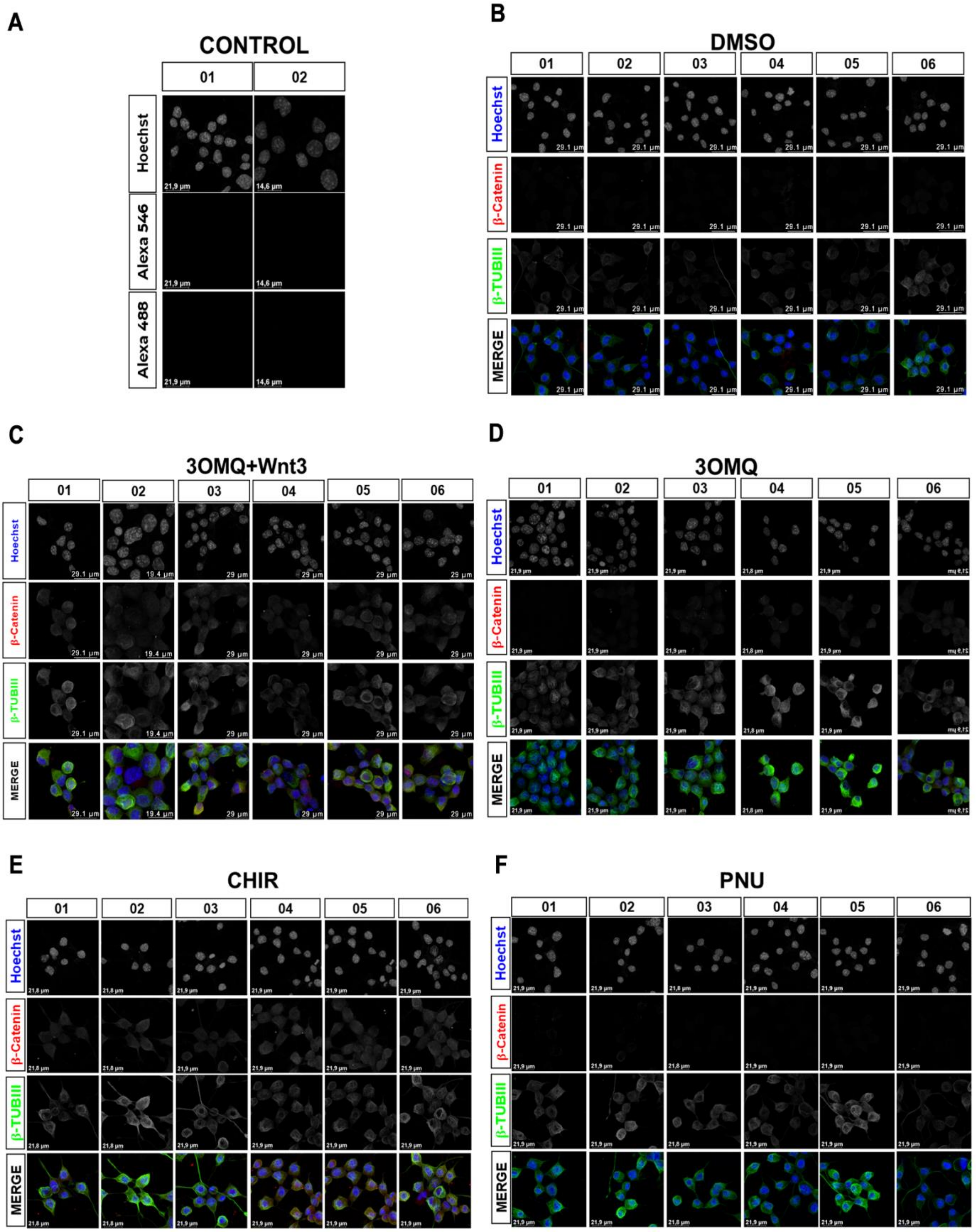

Figure S1. Representative image fields of the immunofluorescence assay used for nuclear β-catenin quantification. Confocal images were acquired using a 63X oil-immersion objective with digital zoom. Six independent confocal fields (01-06) were collected per condition, covering the entire cell population within the coverslip. (A) Sample incubated with secondary antibody only (Alexa Fluor 555 or 488), without primary antibody, to confirm the absence of background fluorescence. (B) Confocal fields of cells that were treated with DMSO. (C) Confocal fields of cells that were treated with 60 μM 3*O*MQ and Wnt3 (10ng/mL). (D) Confocal fields of cells that were treated with 60 μM 3*O*MQ. (E) Confocal fields of cells that were treated with 3 μM CHIR. (F) Confocal fields of cells that were treated with 100 μM PNU.

Supplementary Figure S2

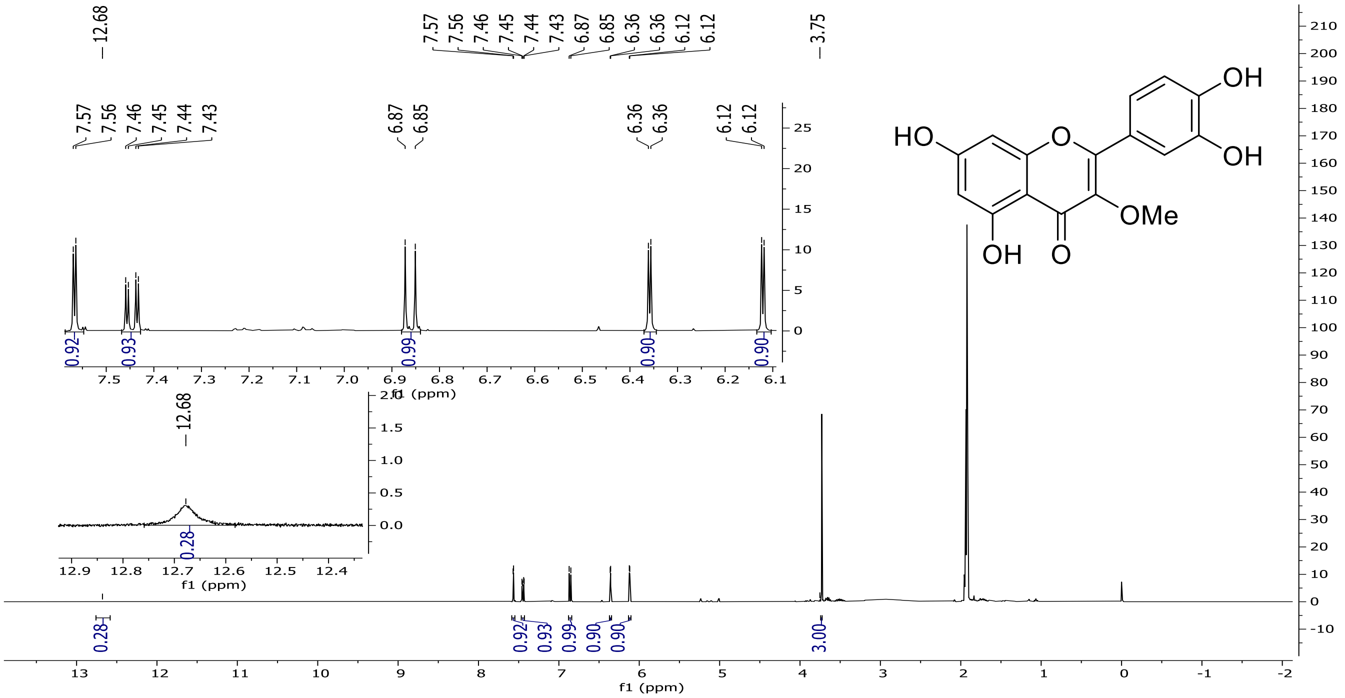

Figure S2. ^{1}H NMR spectrum of 3OMQ. ^{1}H NMR (400 MHz, acetone) δ 12.68 (s, 1H), 7.57 (d, *J* = 2.2 Hz, 1H), 7.45 (dd, *J* = 8.5, 2.2 Hz, 1H), 6.86 (d, *J* = 8.5 Hz, 1H), 6.36 (d, *J* = 2.1 Hz, 1H), 6.12 (d, *J* = 2.1 Hz, 1H), 3.73 (s, 3H).

This data is consistent with that of a natural sample, as reported by Kato *et al.*: ^{1}H NMR (400 MHz, acetone-d_6) δ 7.71 (1H, d, *J* = 1.8 Hz, H-2′), 7.58 (1H, dd, *J* = 8.7 and 1.8 Hz, H-6′), 7.00 (1H, d, *J* = 8.7 Hz, H-5′), 6.50 (1H, d, *J* = 1.8 Hz, H-8), 6.26 (1H, d, *J* = 2.3 Hz, H-6), 3.87 (3H, s, 3-OMe).

KATO, K., NINOMIYA, M., TANAKA, K., KOKETSU, M. Effects of Functional Groups and Sugar Composition of Quercetin Derivatives on Their Radical Scavenging Properties. *J. Nat. Prod.,* **2016**, 79, 7, 1808-1814.

It is noteworthy that 3OMQ is a synthetic compound produced by 2 different routes, which allow precise manipulation of the OH groups.

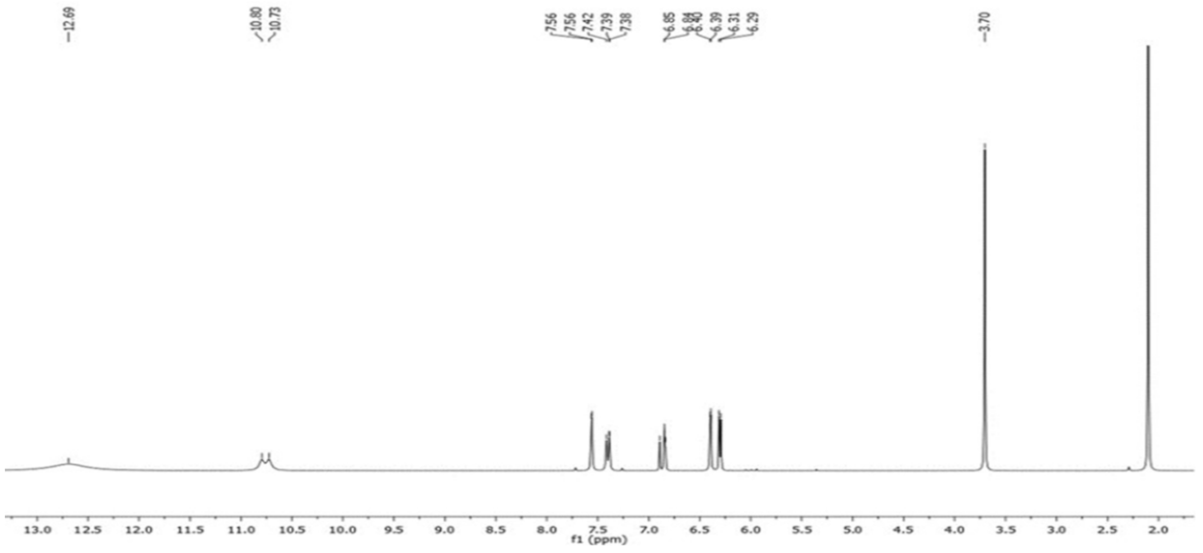

Dados da Literatura: ^1H NMR (300 MHz, acetone-d_6) δ 12,69 (s, 1H), 10,79 (s, 1H), 10,72 (s, 1H), 10,70 (s, 1H) 7.56 (1H, d, J= 1,98 Hz), 7,40 (dd, J=2 and 8,1 Hz), 6,87 (1H, d, J = 8.7 Hz, H-5′), 6.39 (1H, d, J = 1.8 Hz, H-8), 6.30 (1H, d, J = 2.3 Hz, H-6), 3.70 (3H, s, 3-OMe)

ARCHILA, E.G., BAUTISTA, F.R., GARCIA, N., VASQUEZ, J.A.C. A promising blueberry from colombia: antioxidante activity, nutritional and phytochemical composition of Cavendishia nítida (Kunth) A. C.Sm. Cell Press Hliyon, 8, **2022**.

Supplementary Figure S3.

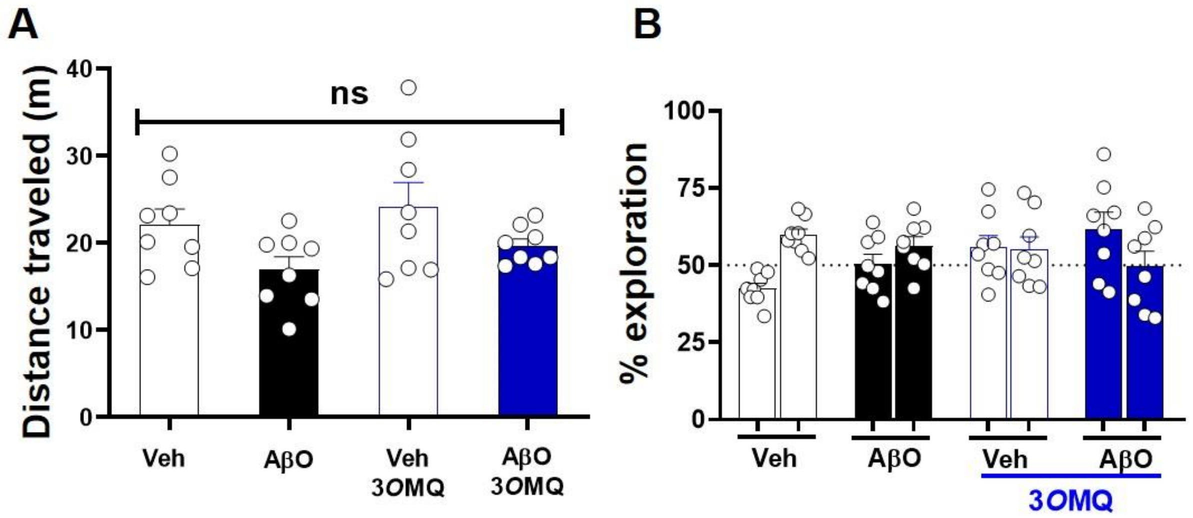

Figure S3. 3-OMQ does not induce changes in locomotor or exploratory behaviors. Female Swiss mice at postnatal day 60 (P60) received 3OMQ or Vehicle (DMSO) via i.c.v., followed 1 hour later by administration of 3 µL of AβO or Vehicle (PBS). (A) Distance traveled in the open field test, performed 24h after i.c.v. treatment and before Novel Object Recognition Task. (B) Exploration towards both objects used during Novel Object Recognition training session. N = 8/group.
